# Supplementary material for: The Pif1 helicase is actively inhibited during meiotic recombination which restrains gene conversion tract length
Source: Nucleic Acids Res. 2021 Apr 6;49(8):4522–33. doi: 10.1093/nar/gkab232 (PMC8096244; doi:10.1093/nar/gkab232)
Supplement: gkab232_Supplemental_Files [file gkab232_supplemental_files.zip › VernekarPif1 paper v6supplmodnochanges.pdf]

Supplementary material

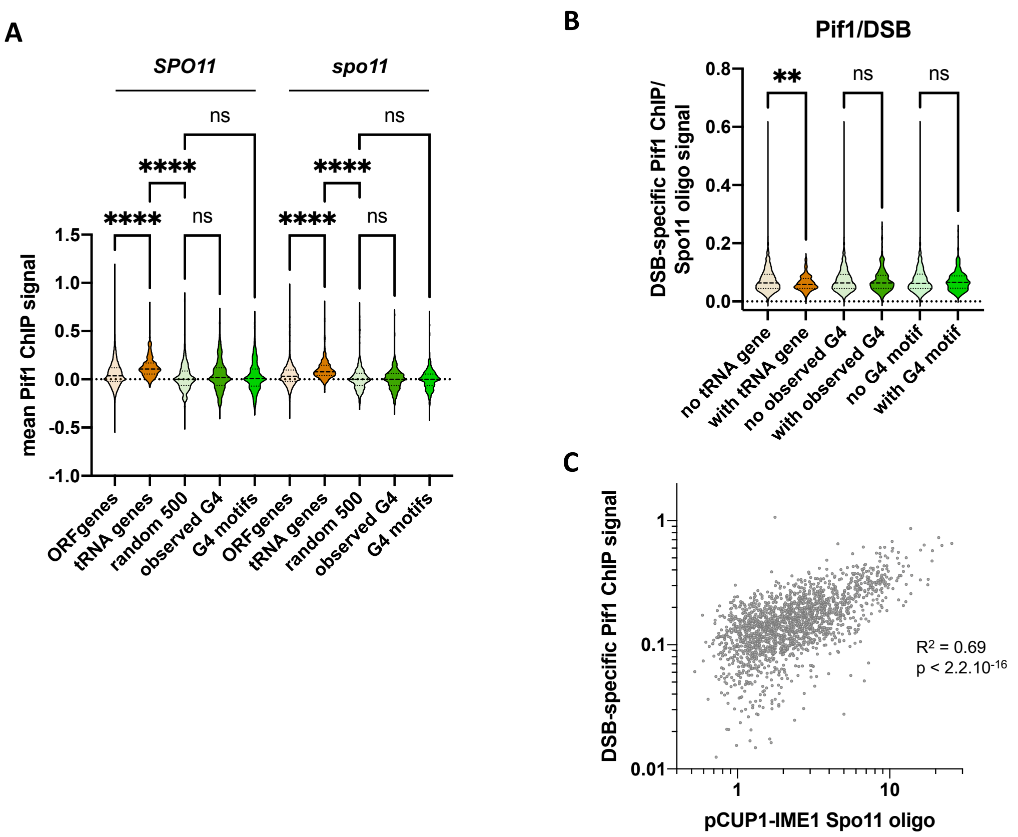

Figure S1: DSB-independent and DSB-specific Pif1 levels close to G4 sequences or tRNA genes

(A) The mean Pif1 ChIP-seq signal from both DSB-proficient (*SPO11*) and -deficient (*spo11*) meiotic cells was background-normalized and measured at 300 pb-long regions spanning the indicated genomic features. ORF genes corresponds to the yeast genome regions spanning from -100 bp to +200 bp from a TSS, to match the tRNA genes profile. Random 500 was generated by randomly selecting 500 genomic regions of 300 bp-long. Observed G4 are according to *in vivo* G4-seq signal measured in (1) (502 sites). G4 motifs are from (2) (668 sites).

(B) The ratio of DSB-specific Pif1 ChIP-seq signal over *pCUP1-IME1* Spo11 oligo signal was computed on the width plus 1 kb on each side of the strongest 2000 Spo11 hotspots located in interstitial regions (more than 20 kb from a centromere and 40 kb from a telomere, 1829 hotspots). These were divided into hotspots containing or not the indicated feature: tRNA genes (96 DSB sites), observed G4 as defined in (1) (211 DSB sites), or G4 motifs as defined in (2) (271 DSB sites).

(A) and (B): violin plots display the median (large dotted line) and the 2 quartiles (dotted lines). See also Table S2 for statistical tests.

(C) DSB-specific Pif1 ChIP-seq signal intensity as a function of DSB (p*CUP1-IME1* Spo11 oligo) signal intensity. The signal intensities at the locations comprised in the strongest 2000 Spo11 hotspots + 1kb on each side were computed for each interstitial hotspot. The Pearson correlation coefficient and the associated p-value are indicated.

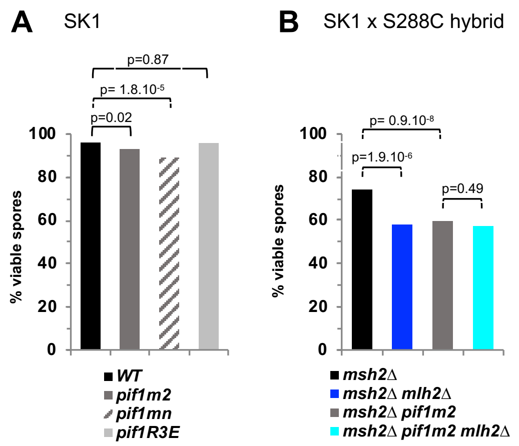

Figure S2: Effect of Pif1 mutants on spore viabilities in SK1 or hybrid strains
(A) Spore viability of SK1 diploids with the indicated relevant genotype.
(B) Spore viability of the SK1 x S288C hybrid used for octad analysis.
See also Table S3

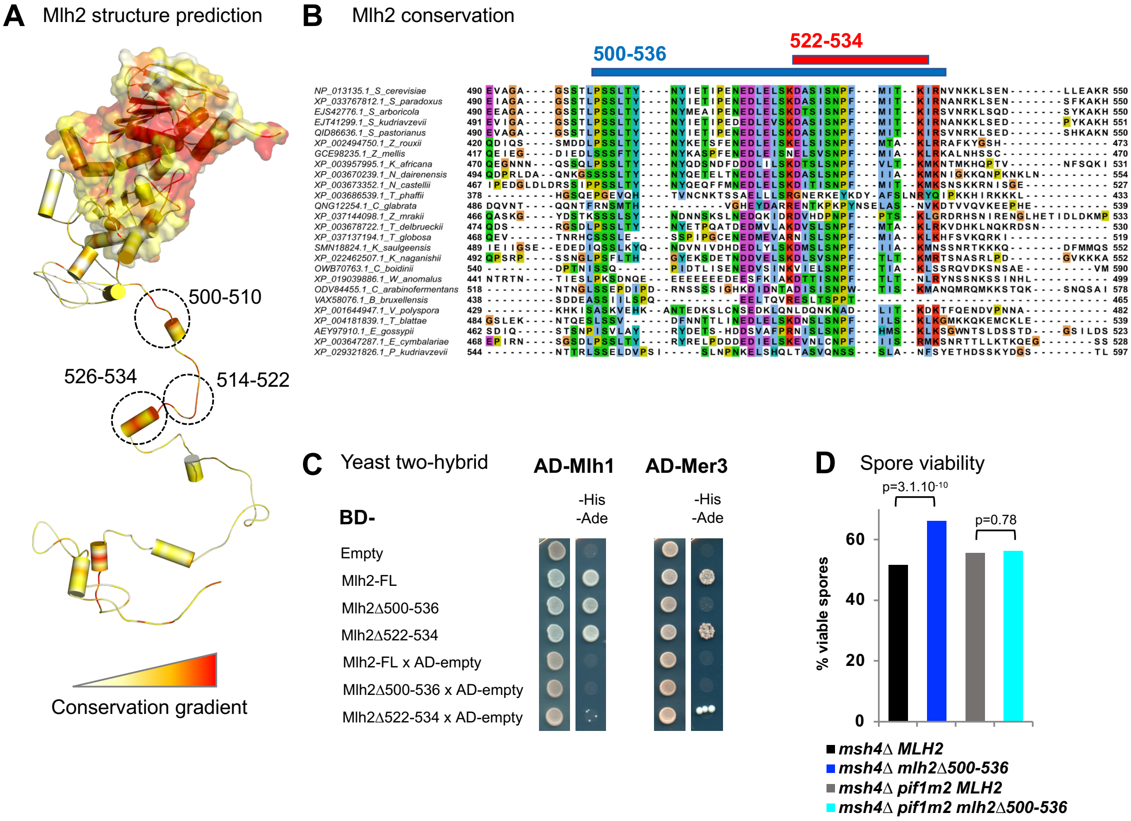

Figure S3: A separation of function mutant of *MLH2* deficient for interaction with Mer3 shows the same phenotype as an *mlh2*Δ mutant for *zmm* spore viability

(A) Model of Mlh2 structure. The candidate regions for interaction with Mer3 are indicated by circles. Conservation is indicated by a color code from red (conserved) to yellow (less conserved).

(B) Alignment of several yeast species in the candidate Mer3 interacting region of Mlh2.

(C) Two-hybrid interactions between Mlh2, Mlh1 and Mer3. The same number of cells of strains expressing the different fusion proteins (BD: Gal4 binding domain; AD: GAL4 activation domain) were plated on minimal media lacking the indicated aminoacids to select for interactions. Growth on -His-Ade medium indicates an interaction. The Mlh1-Mlh2 interaction is used as a positive control. FL: Full length.

(D) Effect of *mlh2Δ500-536* and *pif1m2* mutants on spore viability of *msh4*Δ cells. See also Table S3. Fisher's exact test p-values are indicated.

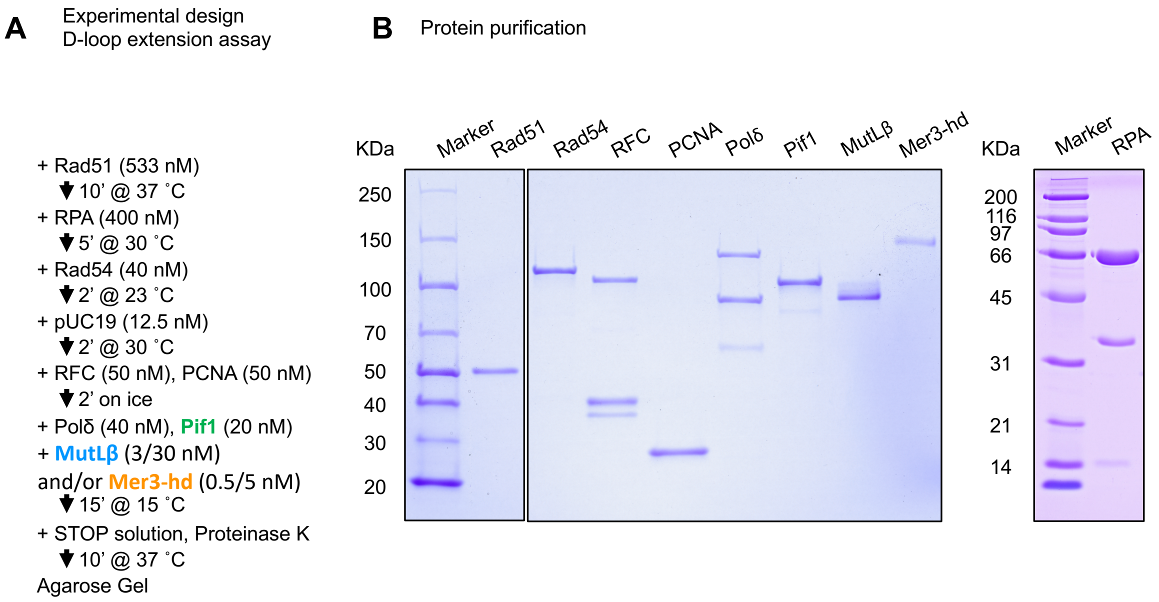

Figure S4: Experimental scheme for the D-loop extension assays and proteins used

(A) Experimental scheme for the D-loop extension assays.

(B) Recombinant proteins were separated by SDS-PAGE on a 4-15% gradient gel and then visualized with Coomassie staining.

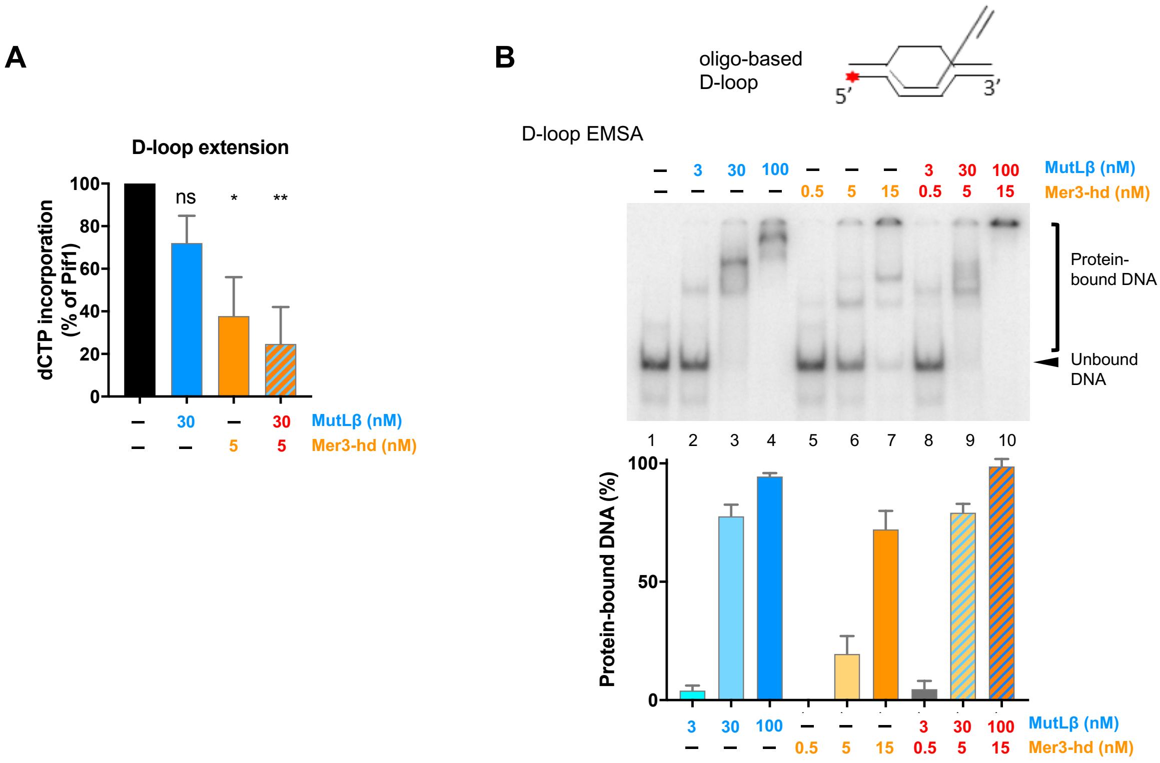

Figure S5: D-loop extension and EMSA in the presence of the Mer3-hd and MutLβ
(A) D-loop extension assay. As in Figure 4A.
(B) D-loop EMSA. The cartoon illustrates the D-loop substrate used in the electrophoretic mobility shift assay (EMSA). Products were resolved by a native gel electrophoresis and quantified. A representative gel is shown. The mean values ± S.E.M. are plotted from three independent experiments.

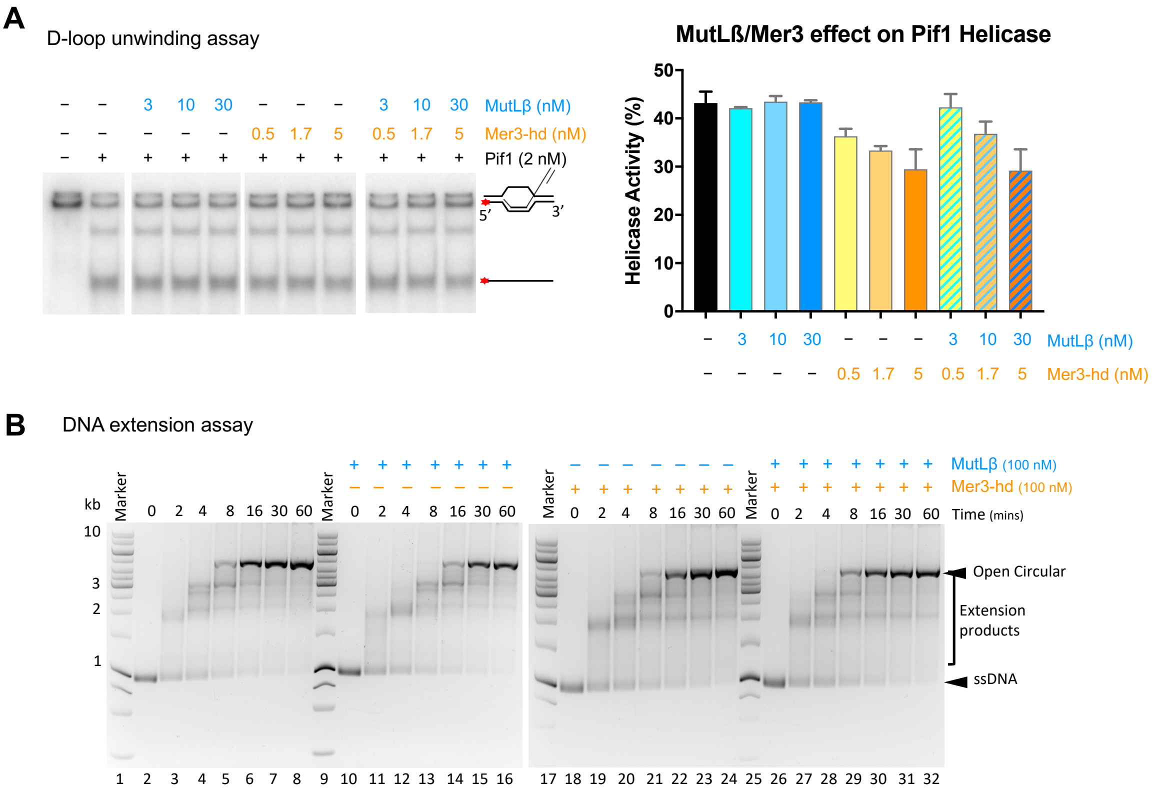

Figure S6: Effect of Mer3 and MutLβ on D-loop unwinding by Pif1 and on DNA extension by Pol δ

(A) D-loop unwinding assay. A representative experiment is shown, and the graph quantitation shows the mean ± S.E.M. of three independent experiments.

(B) DNA extension by Pol δ. Representative gels from the experiments quantified in Figure 4B are shown.

Table S1. Strains used in this study.

All strains are from the SK1 background, unless indicated otherwise.

Figure 1

VBD1907 *a/l ho::hisG/" leu2::hisG/" HIS4::LEU2-(BamH1; +ori)/his4-X::LEU2-(NgoMIV; +ori)-URA3 irt1::KanMX-pCUP1-3HA-IME1/" PIF1-Myc13 ::HphMX/"*

VBD1908 *a/l ho::hisG/" leu2::hisG/" HIS4::LEU2-(BamH1; +ori)/his4-X::LEU2-(NgoMIV; +ori)-URA3 irt1::KanMX-pCUP1-3HA-IME1/" mlh2Δ ::HphMX/" PIF1-Myc13 ::HphMX/"*

VBD1930 *a/l ho::hisG/" leu2::hisG/" HIS4::LEU2-(BamH1; +ori)/his4-X::LEU2-(NgoMIV; +ori)-URA3 irt1::KanMX-pCUP1-3HA-IME1/" spo11Y135F-His6Flag3 ::NatMX/" PIF1-Myc13 ::HphMX/"*

Figure 2

B-C

VBD-HY1 BLY107 (S288c)/BLY114 (SK1) *msh2Δ::HphMX/"* (3)

VBD-HY2 VBD-HY1 *mlh2Δ::KanMX/"* (3)

VBD-HY8 VBD-HY1 *pif1m2/"*

VBD-HY9 VBD-HY1 *pif1m2/" mlh2Δ::KanMX/"*

VBD-HY10 VBD-HY1 *pif1R3E/" mlh2Δ::KanMX/"*

D

VBD1676 *a/l ho::hisG/" leu2::hisG/" ura3/" his4-X::LEU2-(NgoMIV; +ori)-URA3/HIS4::LEU2-(BamH1; +ori) msh4Δ::HphMX/"*

VBD1682 *a/l ho::hisG/" leu2::hisG/" ura3/" his4-X::LEU2-(NgoMIV; +ori)-URA3/HIS4::LEU2-(BamH1; +ori) msh4Δ::HphMX/" mlh2Δ::HphMX/"*

VBD1820 *a/l ho::hisG/" leu2::hisG/" ura3/" HIS4::LEU2-(BamH1; +ori)/his4-X::LEU2-(NgoMIV; +ori)-URA3 msh4Δ::HphMX/" pif1m2/"*

VBD1818 *a/l ho::hisG/" leu2::hisG/" ura3/" HIS4::LEU2-(BamH1; +ori)/his4-X::LEU2-(NgoMIV; +ori)-URA3 msh4Δ::HphMX/" mlh2Δ::HphMX/" pif1m2/"*

VBD2270 *a/l ho::hisG/" leu2::hisG/" ura3/" HIS4::LEU2-(BamH1; +ori)/his4-X::LEU2-(NgoMIV; +ori)-URA3 msh4Δ::HphMX/" pCLB2-HA3-pif1::KanMX/"*

VBD2271 *a/l ho::hisG/" leu2::hisG/" ura3/" HIS4::LEU2-(BamH1; +ori)/his4-X::LEU2-(NgoMIV; +ori)-URA3 msh4Δ::HphMX/" mlh2Δ::HphMX/" pCLB2-HA3-pif1::KanMX/"*

VBD2051 *a/l ho::hisG/" leu2::hisG/" ura3/" his4-X::LEU2-(NgoMIV; +ori)-URA3/HIS4::LEU2-(BamH1; +ori) msh4Δ::HphMX/" pif1R3E/"*

VBD2050 *a/l ho::hisG/" leu2::hisG/" ura3/" his4-X::LEU2-(NgoMIV; +ori)-URA3/HIS4::LEU2-(BamH1; +ori) msh4Δ::HphMX/" mlh2Δ::HphMX/" pif1R3E/"*

E

VBD1907 *a/l ho::hisG/" leu2::hisG/" HIS4::LEU2-(BamH1; +ori)/his4-X::LEU2-(NgoMIV; +ori)-URA3 irt1::KanMX-pCUP1-3HA-IME1/" PIF1-Myc13 ::HphMX/"*

VBD2213 *a/l ho::hisG/" leu2::hisG/" HIS4::LEU2-(BamH1; +ori)/his4-X::LEU2-(NgoMIV; +ori)-URA3 irt1::KanMX-pCUP1-3HA-IME1/" pif1R3E-Myc13 ::HphMX/"*

Figure 3

A

VBD1875 *a/l ho::hisG/" leu2::hisG/" HIS4::LEU2-(BamH1; +ori)/his4-X::LEU2-(BamHI; +ori)-URA3 irt1::KanMX-pCUP1-3HA-IME1/"*

VBD2119 *a/l ho::hisG/" leu2::hisG/" HIS4::LEU2-(BamH1; +ori)/his4-X::LEU2-(BamHI; +ori)-URA3 irt1::KanMX-pCUP1-3HA-IME1/" MER3-TAP ::URA3/"*

B

VBD1911 *a/l ho::hisG/" leu2::hisG/" HIS4::LEU2-(BamH1; +ori)/his4-X::LEU2-(NgoMIV; +ori)-URA3 irt1::KanMX-pCUP1-3HA-IME1/" MER3-His6Flag3 ::NatMX/"*

VBD2146 *a/l ho::hisG/" leu2::hisG/" HIS4::LEU2-(BamH1; +ori)/his4-X::LEU2-(NgoMIV; +ori)-URA3 irt1::KanMX-pCUP1-3HA-IME1/" MER3-His6Flag3 ::NatMX/" PIF1-TAP ::URA3*

VBD2147 *a/l ho::hisG/" leu2::hisG/" HIS4::LEU2-(BamH1; +ori)/his4-X::LEU2-(NgoMIV; +ori)-URA3 irt1::KanMX-pCUP1-3HA-IME1/" MER3-His6Flag3 ::NatMX/" RFC1-TAP ::URA3*

C

VBD1907 *a/l ho::hisG/" leu2::hisG/" HIS4::LEU2-(BamH1; +ori)/his4-X::LEU2-(NgoMIV; +ori)-URA3 irt1::KanMX-pCUP1-3HA-IME1/" PIF1-Myc13 ::HphMX/"*

VBD2047 *a/l ho::hisG/" leu2::hisG/" HIS4::LEU2-(BamH1; +ori)/his4-X::LEU2-(BamHI; +ori)-URA3 irt1::KanMX-pCUP1-3HA-IME1/" PIF1-Myc13 ::HphMX/" MER3-TAP ::URA3/"*

Figure S2

A

VBD1311 *a/l ho::hisG/" leu2::hisG/" ura3/" HIS4::LEU2-(BamH1; +ori)/his4-X::LEU2-(NgoMIV; +ori)-URA3*

VBD1819 *a/l ho::hisG/" leu2::hisG/" ura3/" HIS4::LEU2-(BamH1; +ori)/his4-X::LEU2-(NgoMIV; +ori)-URA3 pif1m2/"*

VBD2269 *a/l ho::hisG/" leu2::hisG/" ura3/" HIS4::LEU2-(BamH1; +ori)/his4-X::LEU2-(NgoMIV; +ori)-URA3 pCLB2-HA3-pif1::KanMX/"*

VBD2049 *a/l ho::hisG/" leu2::hisG/" ura3/" his4-X::LEU2-(NgoMIV; +ori)-URA3/HIS4::LEU2-(BamH1; +ori) pif1R3E/"*

B

VBD-HY1 BLY107 (S288c)/BLY114 (SK1) *msh2Δ::HphMX/"* (3)

VBD-HY2 VBD-HY1 *mlh2Δ::KanMX/"* (3)

VBD-HY8 VBD-HY1 *pif1m2/"*

VBD-HY9 VBD-HY1 *pif1m2/" mlh2Δ::KanMX/"*

Figure S3

VBD2272 *a/l ho::hisG/" leu2::hisG/’’ ura3/” his4-X::LEU2-(NgoMIV; +ori)-URA3/HIS4::LEU2-(BamH1; +ori) msh4∆::HphMX/” MLH2-Myc13::HphMX/”*

VBD2267 *a/l ho::hisG/" leu2::hisG/’’ ura3/” his4-X::LEU2-(NgoMIV; +ori)-URA3/HIS4::LEU2-(BamH1; +ori) msh4∆::HphMX/” mlh2∆500-536-Myc13::HphMX/”*

VBD2273 *a/l ho::hisG/" leu2::hisG/’’ ura3/” HIS4::LEU2-(BamH1; +ori)/his4-X::LEU2-(NgoMIV; +ori)-URA3 msh4∆::HphMX/” MLH2-Myc13::HphMX/” pif1m2/”*

VBD2268 *a/l ho::hisG/" leu2::hisG/’’ ura3/” HIS4::LEU2-(BamH1; +ori)/his4-X::LEU2-(NgoMIV; +ori)-URA3 msh4∆::HphMX/” mlh2∆500-536-Myc13::HphMX/” pif1m2/”*

Table S2: Pif1 signal at the indicated genomic features

Figure S1A

	Mean 1	Mean 2	Adjusted P Value (Games-Howell's multiple comparisons test)
SPO11 ORFgenes vs. tRNA genes	0.05523	0.1292	<0.0001
SPO11 tRNA genes vs. random 500	0.1292	0.01819	<0.0001
SPO11 random 500 vs. observed G4	0.01819	0.04313	0.2546
SPO11 random 500 vs. G4 motifs	0.01819	0.03015	0.9429
spo11 ORFgenes vs. tRNA genes	0.04919	0.1024	<0.0001
spo11 tRNA genes vs. random 500	0.1024	0.01735	<0.0001
spo11 random 500 vs. observed G4	0.01735	0.004572	0.8797
spo11 random 500 vs. G4 motifs	0.01735	0.003034	0.7176

Figure S1B

	Mean 1	Mean 2	Adjusted P Value (Games-Howell's multiple comparisons test)
no tRNA gene vs. with tRNA gene	0.07409	0.06323	0.0031
no observed G4 vs. with observed G4	0.07373	0.07176	0.984
no G4 motif vs. with G4 motif	0.07398	0.07085	0.777

Table S3: Spore viabilities

Strain name	Genotype	Tetrad type					Spore viability (%)	Number of tetrads	Fisher Test p-Value
		4 sp	3 sp	2 sp	1 sp	0 sp			
VBD1311	WT (3)	131	9	7	0	0	96.1	147	vs WT
VBD1819	*pif1m2*	104	21	6	0	1	93.0	132	0.0239
VBD2269	*pif1mn*	69	24	7	1	1	89.0	102	1.8E-05
VBD2049	*pif1R3E*	116	10	2	0	2	95.8	130	0.87
VBD1676	*msh4Δ*	53	8	43	10	104	38.1	218	vs *msh4Δ*
VBD1682	*msh4Δ mlh2Δ* (3)	35	5	29	2	29	53.8	100	2.1E-07
VBD1820	*msh4Δ pif1m2*	27	10	31	10	52	40.4	130	vs *msh4Δ pif1m2*
VBD1818	*msh4Δ pif1m2 mlh2Δ*	36	6	29	10	49	44.2	130	0.23
VBD2270	*msh4Δ pif1mn*	20	13	24	5	40	42.2	102	vs *msh4Δ pif1mn*
VBD2271	*msh4Δ pif1mn mlh2Δ*	25	11	17	6	42	42.08	101	0.89
VBD2051	*msh4Δ pif1R3E*	40	6	45	13	103	33.9	207	vs *msh4Δ pif1R3E*
VBD2050	*msh4Δ pif1R3E mlh2Δ*	50	7	41	9	101	37.5	208	0.13
VBD2272	*msh4Δ MLH2-Myc*	77	6	47	8	69	51.7	207	*vs msh4Δ MLH2-Myc*
VBD2267	*msh4Δ mlh2Δ500-536-Myc*	130	11	59	9	48	66.1	257	3.1E-10
VBD2273	*msh4Δ pif1m2 MLH2-Myc*	70	30	39	8	58	55.6	205	*vs msh4Δ pif1m2 MLH2-Myc*
VBD2268	*msh4Δ pif1m2 mlh2Δ500-536-Myc*	89	31	62	10	67	56.3	259	0.78
HY1	*msh2Δ* (3)	48	33	26	13	0	74.2	120	vs *msh2Δ*
HY2	*msh2Δ mlh2Δ* (3)	16	30	30	13	9	57.9	98	5.5E-07
HY8	*msh2Δ pifm2*	32	19	40	16	11	59.5	118	1.9E-06
HY9	*msh2Δ mlh2Δ pif1m2*	21	27	27	19	10	57.2	104	9.9E-08

Table S4: List of all recombination events detected from octad analyses in the reference and in the mutant strains (Excel spreadsheet)

Table S5: List of the proteins significantly enriched in the Mer3-TAP pull downs (Excel spreadsheet)

Supplemental references

1. Marsico, G., Chambers, V.S., Sahakyan, A.B., McCauley, P., Boutell, J.M., Antonio, M.D. and Balasubramanian, S. (2019) Whole genome experimental maps of DNA G-quadruplexes in multiple species. *Nucleic Acids Res*, **47**, 3862-3874.

2. Capra, J.A., Paeschke, K., Singh, M. and Zakian, V.A. (2010) G-quadruplex DNA sequences are evolutionarily conserved and associated with distinct genomic features in Saccharomyces cerevisiae. *PLoS Comput Biol*, **6**, e1000861.
3. Duroc, Y., Kumar, R., Ranjha, L., Adam, C., Guerois, R., Md Muntaz, K., Marsolier-Kergoat, M.C., Dingli, F., Laureau, R., Loew, D. *et al.* (2017) Concerted action of the MutLbeta heterodimer and Mer3 helicase regulates the global extent of meiotic gene conversion. *Elife*, **6**, e21900.
